# Tracing feline herpesvirus-1 exposure in domestic cats from Namibia: associations with neutralising antibodies, vaccination, clinical signs, and retroviral status

**DOI:** 10.1007/s11259-026-11446-3

**Published:** 2026-08-05

**Authors:** Lourens de Villiers, Simone Pulsoni, Barbara Bonfini, Lauren M. Coetzee, Ellini Hamunyela, Mari de Villiers, Eugenia Ciarrocchi, Alessandra Leone, Liana Teodori, Umberto Molini

**Affiliations:** 1 Department of Companion Animal Clinical Studies, Faculty of Veterinary Science, University of Pretoria, Private Bag X04, Onderstepoort, South Africa; 2 Department of Companion Animal Clinical Studies, Faculty of Health Sciences and Veterinary Medicine, University of Namibia, Neudamm Campus, Private Bag 13301, Windhoek, Namibia; 3 Istituto Zooprofilattico Sperimentale dell’Abruzzo e del Molise, Teramo, 64100 Italy; 4 Central Veterinary Laboratory, 24 Goethe Street, Private Bag 18137, Windhoek, Namibia; 5 Rhino Park Veterinary Clinic, 54 Rhino Street, PO Box 50533, Windhoek, Namibia

**Keywords:** FHV-1, Feline, Serology, VNT, Immunocompetence, FIV, FeLV

## Abstract

Feline herpesvirus type 1 (FHV-1) is a major cause of upper respiratory and ocular disease in domestic cats and remains widely distributed worldwide. However, information on FHV-1 exposure and neutralising antibody responses in cats from Namibia is limited. This study assessed FHV-1 neutralising antibody prevalence in a convenience sample of 236 domestic cats sampled across 15 towns in eight regions of Namibia. Neutralising antibodies were detected in 18/236 (8%) cats (95% CI: 4.88–11.73%). Seropositive animals were identified only in Erongo, Khomas, and Hardap regions, with the highest regional seroprevalence observed in Erongo. Seropositivity was uncommon in both vaccinated and unvaccinated cats and was not significantly associated with vaccination status, feline immunodeficiency virus antibody status, or feline leukaemia virus antigen status. A significant association was identified between seropositivity and oculonasal discharge (*p* < 0.001). Overall, the findings indicate low FHV-1 neutralising antibody prevalence within the sampled population rather than the wider domestic cat population of Namibia, and suggest that detectable neutralising antibodies were observed predominantly in unvaccinated cats, although interpretation is limited by the small number of vaccinated animals and the absence of detailed vaccination histories.

**Supplementary Information:**

The online version contains supplementary material available at https://doi.org/10.1007/s11259-026-11446-3.

## Introduction

Feline herpesvirus type 1 (FHV-1) is a highly contagious alphaherpesvirus and the principal causative agent of feline viral rhinotracheitis, a disease primarily affecting the upper respiratory tract and ocular tissues of felids (Gaskell et al. 2007; Davison et al. 2009). Transmission occurs mainly through direct or indirect contact with infected secretions. Following primary infection, the virus establishes lifelong latency with periodic reactivation, facilitating sustained circulation within feline populations (Yang et al. 2025). Despite the availability of therapeutic and preventive measures, FHV-1 remains widely distributed and continues to pose a challenge to feline health.

The immunological status of domestic cats, particularly the presence of virus-specific neutralising antibodies, plays an important role in susceptibility to infection and clinical disease expression. Serological evidence indicates widespread natural exposure to FHV-1, with cats developing variable levels of humoral immunity (Gaskell et al. 2007; Lee et al. 2019). However, detection of neutralising antibodies reflects prior exposure rather than active infection, and seropositive cats may continue to harbour latent virus with intermittent viral shedding, particularly under conditions of stress or immunosuppression.

Vaccination remains an important component of FHV-1 prevention and control. Available vaccines are primarily based on either inactivated or modified-live virus formulations. Modified-live vaccines generally induce stronger humoral and cellular immune responses than inactivated vaccines, although both vaccine types reduce clinical disease and viral shedding without preventing establishment of viral latency. Furthermore, protection against FHV-1 is not solely dependent on detectable neutralising antibody titres (Squires et al. 2024). Consequently, FHV-1 may persist even in vaccinated populations, underscoring the importance of continued epidemiological surveillance and regional seroprevalence studies to better understand patterns of exposure and population immunity.

Across southern Africa, the interpretation of vaccine-associated serological findings remains challenging because humoral responses vary according to vaccine formulation, vaccine history, time since vaccination, and host immune responses, while protective immunity is not solely dependent on detectable neutralising antibody titres (Scherk et al. 2013; Squires et al. 2024). Furthermore, detailed information regarding vaccination practices and vaccine utilisation in domestic cats from Namibia remains unavailable.

In Namibia, the epidemiology of infectious diseases in domestic cats is influenced by diverse ecological and socio-environmental conditions. Many cats occur in rural or peri-urban settings with variable access to veterinary care and vaccination, and a substantial proportion of the population is likely unvaccinated (De Villiers et al. 2025). Despite the close association between domestic cats, wildlife, and human settlements in many parts of the country, information on FHV-1 exposure and neutralising antibody responses in Namibian cats remains limited.

Published information describing FHV-1 epidemiology in southern Africa remains sparse, with available studies focusing predominantly on wildlife species rather than domestic cats. Early serological surveys demonstrated widespread herpesvirus exposure in free-ranging lions in South Africa (Spencer 1991), while clinical FHV-1 infection has been described in semi-captive cheetahs (Van Vuuren et al. 1999). Subsequent serological investigations identified evidence of FHV-1 exposure in free-ranging cheetahs from Namibia (Munson et al. 2004; Thalwitzer et al. 2010) and, more recently, in free-ranging servals from South Africa (Loock et al. 2021). By contrast, comparable population-level epidemiological studies investigating FHV-1 exposure in domestic cats from southern Africa remain limited, particularly those employing virus neutralisation assays. Consequently, important gaps remain regarding regional seroprevalence, associated epidemiological factors, and patterns of naturally acquired immunity among domestic cats. This study aimed to assess FHV-1 neutralising antibody prevalence in domestic cats from eight regions of Namibia while considering age, sex, vaccination status, clinical signs, and previously reported retroviral status.

## Materials and methods

### Study site and design

**Fig. 1:**
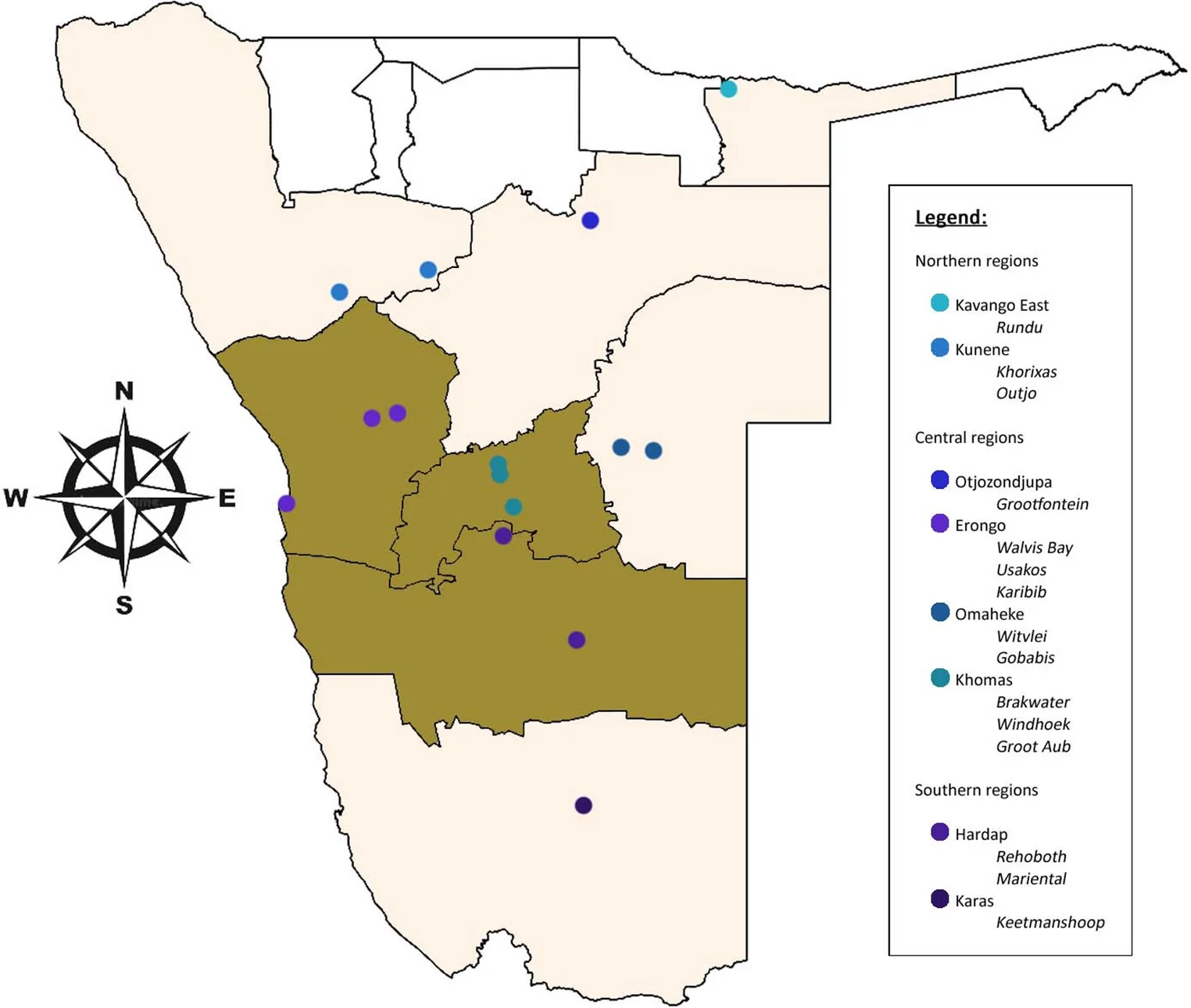
Origin sites of cats across eight regions of Namibia

Serum samples from 236 cats across 15 towns in eight regions of Namibia were analysed (Fig. 1). Overall, sampling sites spanned the northern, central, and southern parts of the country. The majority of locations were rural, with low population density and limited access to private veterinary services due to financial constraints. Serum samples were initially collected through non-probability convenience sampling during routine diagnostic procedures for cats presented to the mobile animal clinic of the Veterinary Academic Hospital, University of Namibia, between April and October 2022. Stored serum samples were subsequently used for secondary analysis in this study to assess the seroprevalence of FHV-1 across these regions. Additionally, metadata on sex, age, vaccination status, and clinical signs recorded at presentation were obtained from the clinical records generated during the mobile veterinary clinic consultations. Notably, findings should be interpreted in the context of convenience sampling, since enrolment depended on presentation to the mobile clinic.

### Cell culture and virus preparation

Crandell-Rees Feline Kidney (CRFK) cells were maintained in Eagle’s Minimum Essential Medium (MEM; Sigma-Aldrich, USA), supplemented with 10% foetal bovine serum (FBS; Euroclone, Italy) at 37 °C in a humidified atmosphere containing 5% CO₂. For virus propagation, CRFK monolayers grown to approximately 90% confluence in 10 cm culture dishes were infected with a double-reporter FHV-1 strain and incubated in MEM, supplemented with 2% FBS. At 72 h post-infection, culture supernatants containing progeny virus were collected. The recovered virus was first titrated and subsequently confirmed as FHV-1 by quantitative real-time polymerase chain reaction, then aliquoted and stored at -80 °C until use. All procedures involving infectious virus were performed in a biosafety level 2 laboratory.

### Virus neutralisation test

Neutralising antibodies against FHV-1 were detected using a virus neutralisation test (VNT) in microtiter plates. Prior to testing, serum samples were heat-inactivated at 56 °C for 30 min. Sera were serially diluted from 1:2 to 1:256 and mixed with an equal volume of FHV-1 reference virus containing 100 TCID₅₀ (tissue culture infectious dose). After 1 h of incubation at 37 °C in a humidified incubator with 5% CO₂, approximately 10⁴ CRFK cells were added to each well. Positive and negative control sera, as well as cell and virus controls (ATCC® Number: VR-636™ Lot Number: 59405823), were included in each assay. Positive and negative serum controls were laboratory-maintained reference sera routinely used for assay validation. All samples were tested in duplicate. Following incubation for 3–5 days, plates were examined for cytopathic effect (CPE). A sample was considered positive when > 90% inhibition of CPE was observed at a serum dilution of 1:2. The final antibody titre was defined as the highest serum dilution showing > 90% inhibition of CPE. All samples were tested in duplicate for confirmation. Only samples positive on both initial and duplicate testing were classified as seropositive; all remaining samples were classified as negative.

### Statistical analysis

Neutralising antibody prevalence of FHV-1 and population metadata, including region, town, sex, age group, vaccination status, clinical signs, feline immunodeficiency virus (FIV) antibody status, and feline leukaemia virus (FeLV) antigen status, were summarised as proportions in tables. Overall and regional prevalence estimates were accompanied by 95% confidence intervals (CI) calculated using the Wilson score method. Associations between seropositivity and categorical variables, including vaccination status, clinical signs, FeLV antigen status, and FIV antibody status, were assessed using Fisher’s exact test. Odds ratios (OR) with 95% CI were calculated for selected two-by-two comparisons. Proportions were expressed as percentages, and statistical significance was set at *p* < 0.05. Given the limited number of vaccinated and seropositive cats, analyses involving these subgroups were considered exploratory. Multivariable logistic regression was not considered sufficiently powered and no adjustment for multiple comparisons was applied, because only 18 cats were seropositive and only 23 cats had a recorded vaccination history.

## Results

### Seroprevalence and geographic distribution

Virus neutralisation testing identified an overall seroprevalence of 8% (95% CI: 4.88**–**11.73%) for neutralising antibodies. Antibody titres among seropositive cats ranged from 1:4 to 1:256, although the assay positivity threshold was defined as inhibition at a dilution of 1:2. Seropositive cats were detected only in three of the eight investigated regions (Fig. 1). The highest regional seroprevalence was observed in Erongo (23%), followed by Khomas (16%) and Hardap (3%), while no seropositive animals were identified in Karas, Kavango-East, Kunene, Omaheke, or Otjozondjupa (Table 1). However, the absence of detectable antibodies in a particular town or region should not be interpreted as evidence of absence of previous FHV-1 exposure or viral circulation, since regional sample sizes were uneven and, in some locations, very small. Individual-level metadata, including clinical information and antibody titres, are provided in Supplementary Dataset S1.

**Table 1:** Neutralising antibody seroprevalence of FHV-1 in domestic cats across eight regions of Namibia

Region	Town		Number of cats	FHV-1 seropositive cats		
				*N*	Town (%)	Region (%) (95% CI)
Erongo	Karibib		4	0	0	23.08 (12.65–38.34)
	Usakos		10	0	0	
	Walvis Bay		25	9	36.0	
Hardap	Mariental		22	0	0	2.94 (0.52–14.92)
	Rehoboth		12	1	8.33	
Karas	Keetmanshoop		26	0	0	0 (0.0–12.87)
Kavango-East	Rundu		39	0	0	0 (0.0–8.97)
Khomas	Windhoek		23	2	8.70	15.69 (8.17–28.01)
	Groot Aub		5	1	20.0	
	Brakwater		23	5	21.74	
Kunene	Khorixas		4	0	0	0 (0.0–32.44)
	Outjo		4	0	0	
Omaheke	Witvlei		15	0	0	0 (0.0–12.06)
	Gobabis		13	0	0	
Otjozondjupa	Grootfontein		11	0	0	0 (0.0–25.88)
Total			**236**	**18**	**7.63 (4.88–11.73)**	

### Population characteristics

A total of 236 feline serum samples were included in the analysis, with a near-equal distribution between male (50.4%) and female (49.6%) cats and predominantly composed of animals older than one year (69%). Similar demographic patterns were observed within both the vaccinated and unvaccinated groups, although vaccinated cats represented a substantially smaller proportion (10%) of the study population (Table 2).

**Table 2:** Signalment and vaccination status among the sampled feline population

	Category	Total *N* (%)	Vaccinated *N* (%)	Unvaccinated * N *(%)
Individuals (*N*)		236	23	213
Sex	Male	119 (50.42)	10 (43.48)	109 (51.17)
	Female	117 (49.58)	13 (56.52)	104 (48.83)
Age	< 1 year	74 (31.36)	7 (30.43)	67 (31.46)
	> 1 year	162 (68.64)	16 (69.57)	146 (68.54)

### Associations with seropositivity

Seropositivity was uncommon in both vaccinated (4%) and unvaccinated (8%) cats, and no significant association was detected between vaccination status and seropositivity. Detectable neutralising antibodies were identified in only one vaccinated animal. A significant association was observed between seropositivity and oculonasal discharge and such cats had substantially greater odds of detectable FHV-1 neutralising antibodies than cats without discharge (OR = 10.10; 95% CI: 3.64–28.07; *p* < 0.001). Vaccinated cats had lower odds of detectable FHV-1 neutralising antibodies than unvaccinated cats, although the association was not statistically significant (OR = 0.52; 95% CI: 0.07–4.13; *p* = 1.000). No significant associations were detected between seropositivity and other recorded clinical variables, including pyrexia, anorexia, lymphadenopathy, submandibular enlargement, respiratory distress, or weight loss (all *p* > 0.05). Likewise, no associations were detected between seropositivity and FIV antibody positivity (OR = 1.37; 95% CI: 0.16–11.43) or FeLV antigen status (OR = 0.40; 95% CI: 0.13–1.25) (Table 3).

**Table 3:** Associations between FHV-1 seropositivity and vaccination, clinical, and retroviral variables

	Category	Total *N* (%)	FHV-1 VNT positive *N* (%)	FHV-1 VNT negative *N* (%)	*p*-value	OR (95% CI)
Individuals (*N*)		236	18	218		
Vaccination status	Vaccinated	23 (9.75)	1 (4.35)	22 (95.65)	1.0	0.52 (0.07–4.13)
	Unvaccinated	213 (90.25)	17 (7.98)	196 (92.02)		
Oculonasal discharge	Present	34 (14.41)	10 (29.41)	24 (70.59)	**< 0.001**	10.10 (3.64–28.07)
	Absent	202 (85.59)	8 (3.96)	194 (96.04)		
*FeLV status	Positive	95 (40.25)	4 (4.21)	91 (95.79)	0.135	0.40 (0.13–1.25)
	Negative	141 (59.75)	14 (9.93)	127 (90.07)		
*FIV status	Positive	10 (4.24)	1 (10.0)	9 (90.0)	0.555	1.37 (0.16–11.43)
	Negative	226 (95.76)	17 (7.52)	209 (92.48)		

Fisher’s exact significance (2-sided): *p* < 0.05 = statistical significance; * (De Villiers et al. 2025).

## Discussion

Neutralising antibodies against FHV-1 were detected at a relatively low overall seroprevalence (8%) in domestic cats sampled across multiple regions of Namibia. Comparatively, the overall seroprevalence is consistent with select international studies (Ravicini et al. 2016; Amoroso et al. 2022). However, it is generally lower than other global findings which range between 16 and 84% (Nakamura et al. 1999; Lappin et al. 2002; Fernandez et al. 2017; Cottingham et al. 2022). Detection of neutralising antibodies reflects previous exposure and immune recognition rather than active infection at the time of sampling (Lee et al. 2019). Therefore, the comparatively low seroprevalence observed in the present study may reflect a combination of reduced transmission opportunities, waning antibody titres, assay limitations, and characteristics within the sampled mobile-clinic population. Importantly, seronegativity should not be interpreted as evidence of absence of previous FHV-1 infection, since latently infected cats may have antibody titres below the assay detection threshold while retaining the capacity for viral reactivation and shedding.

The geographic distribution of seropositive animals suggested marked regional variation in exposure patterns. Higher seroprevalence was observed in Erongo and Khomas, particularly in urban and peri-urban localities such as Walvis Bay, Windhoek, and Brakwater, whereas no seropositive animals were identified in several predominantly rural regions. This pattern may reflect differences in host density and contact structure, as urban centres and coastal settlements typically support larger cat populations, greater numbers of multi-cat households, and increased opportunities for direct transmission of respiratory pathogens. In contrast, the absence of detectable seropositive animals in some regions should not be interpreted as evidence of absence of viral circulation. Given the ability of herpesviruses to establish lifelong latency, combined with uneven regional sampling intensity and small sample numbers in some areas, undetected circulation remains plausible.

No apparent sex-related differences in seropositivity were identified, consistent with the absence of a recognised intrinsic sex predilection for FHV-1 infection. The near-equal sex distribution observed in the cohort suggests that exposure risk in this population was likely influenced more by environmental or management-associated factors than by biological susceptibility. Most cats in the study population were older than one year of age, which may have influenced interpretation of serological findings. Older animals have a greater cumulative probability of previous exposure, while neutralising antibodies may persist following infection. Consequently, seropositivity in this cohort likely reflects historical exposure patterns rather than recent infection dynamics alone. The predominance of adult animals also limits inference regarding primary exposure and early immune responses in younger cats.

Vaccinated cats represented a relatively small proportion of the study population, and only one of the 23 vaccinated cats possessed detectable neutralising antibodies. No significant association was identified between vaccination status and seropositivity, while most seropositive cats were unvaccinated, suggesting that detectable neutralising antibodies in this cohort were more commonly observed among unvaccinated animals than vaccinated animals. This observation should be interpreted cautiously, as current vaccines reduce clinical disease and viral shedding but do not prevent latency or necessarily induce persistent detectable antibody responses in all animals (Magouz et al. 2022). Nevertheless, interpretation of these findings should consider the limited number of vaccinated animals. In addition, detailed vaccination histories, including vaccine formulation, timing of administration, booster status, and vaccine handling conditions, were unavailable. Possible differences in neutralising antibody responses induced by modified-live versus inactivated FHV-1 vaccines could not be evaluated in this cohort, because vaccine formulation was not recorded. Although published studies from neighbouring South Africa provide some insight into vaccination practices among privately-owned cats, comparable information regarding vaccination coverage and commonly used FHV-1 vaccine formulations in Namibia remains unavailable. These factors may influence serological responses and complicate assessment of vaccine-associated immunity under field conditions.

The low frequency of detectable neutralising antibodies among vaccinated cats may reflect waning humoral responses, incomplete vaccination histories, timing since vaccination, vaccine handling conditions, or limitations of antibody-based assessment rather than inadequate vaccine efficacy. Furthermore, FHV-1 vaccines are intended primarily to reduce clinical disease and viral shedding, while protective immunity is not solely dependent on detectable neutralising antibody titres and may involve important cellular immune responses that were not evaluated in the present study (Scherk et al. 2013; Squires et al. 2024).

A significant association was identified between seropositivity and oculonasal discharge, consistent with the recognised role of FHV-1 as a major cause of feline upper respiratory and ocular disease (Gaskell et al. 2007; Davison et al. 2009). Cats with detectable neutralising antibodies were more likely to exhibit oculonasal discharge, potentially reflecting prior infection, recrudescence of latent infection, or repeated exposure within environments conducive to viral transmission (Yang et al. 2025). However, seropositivity alone does not confirm active infection at the time of sampling, and clinical signs observed in seronegative animals may have resulted from other infectious or non-infectious causes. No significant associations were observed between seropositivity and other recorded clinical variables, including pyrexia, anorexia, lymphadenopathy, submandibular enlargement, respiratory distress, or weight loss, which likely reflects the non-specific and multifactorial nature of disease aetiology in many cats.

The interpretation of FHV-1 neutralising antibody responses should also consider the retroviral status previously reported for the same feline cohort. Previous molecular investigations detected FIV nucleic acid in blood samples from a small proportion of cats (1%) (Franzo et al. 2024) while FIV antibodies were recorded in 4% cats (De Villiers et al. 2025). In contrast, FeLV antigen seropositivity was comparatively frequent, being detected in 40% of the feline population (De Villiers et al. 2025). The high FeLV prevalence observed in this cohort likely reflects characteristics of the sampled mobile-clinic population rather than the wider domestic cat population of Namibia and should therefore be interpreted cautiously. No statistically significant associations were detected between seropositivity and FIV antibody status or FeLV antigen status. Since FeLV status was derived from previously reported antigen testing (De Villiers et al. 2025) and not independently re-tested in the present analysis, possible misclassification cannot be fully excluded. Although retroviral infections may impair immune function and increase susceptibility to clinical disease, antibody detection against FHV-1 reflects exposure history rather than disease severity alone. An exploratory observation in the present study was that a proportion of vaccinated cats lacking detectable neutralising antibodies were FeLV antigen-positive. Given the recognised immunomodulatory effects of FeLV infection, including impaired humoral immune responses, the occurrence of FeLV-positive vaccinated cats lacking detectable neutralising antibodies warrants further investigation in larger, targeted cohorts.

Several factors should be considered when interpreting these findings. Sampling was based on convenience recruitment through a mobile veterinary service and therefore may not fully represent the broader domestic cat population of Namibia. Although most sampled cats originated from rural or peri-urban settings where free-roaming husbandry is common, individual-level feeding mode and indoor/outdoor living environment were not recorded, which further limits inference regarding exposure risk. In addition, vaccinated animals comprised only a small proportion of the cohort, limiting statistical power for vaccination-associated analyses. Nonetheless, the study provides preliminary insight into FHV-1 neutralising antibody prevalence and associated epidemiological patterns in domestic cats from multiple regions of Namibia, where information on feline infectious disease exposure remains limited. These findings also contribute baseline information on FHV-1 exposure in domestic cats within a country where interactions among domestic animals, wildlife, and human settlements may influence pathogen transmission dynamics (Thalwitzer et al. 2010).

## Conclusion

This study provides the first regional overview of FHV-1 neutralising antibody prevalence in domestic cats from Namibia. Within the sampled population, overall seroprevalence was low, with evidence of exposure restricted to selected regions and detectable antibodies observed predominantly among unvaccinated cats. However, interpretation of vaccination-associated findings is limited by the small number of vaccinated animals and the absence of detailed vaccination histories. Seropositivity was significantly associated with oculonasal discharge, supporting the recognised role of FHV-1 in feline upper respiratory disease. No significant associations were identified with retroviral status, although the occurrence of FeLV-positive animals among vaccinated cats lacking detectable neutralising antibodies warrants further investigation. Future studies incorporating larger and more geographically representative cohorts, detailed vaccination histories, and longitudinal sampling would improve understanding of FHV-1 epidemiology and immunity in Namibian cats.

## Supplementary Information

Below is the link to the electronic supplementary material.

Supplementary Material 1 (DOCX 25.0 KB)

## Data Availability

Supplementary patient metadata is recorded in supplementary dataset S1. The datasets generated and/or analysed during the current study are also available from the corresponding author upon reasonable request.

## Abbreviations

CPE: Cytopathic effect
CRFK: Crandell-Rees feline kidney
FBS: Foetal bovine serum
FeLV: Feline leukaemia virus
FHV-1: Feline herpesvirus type 1
FIV: Feline immunodeficiency virus
MEM: Minimum essential medium
TCID: Tissue culture infectious dose
VNT: Virus neutralisation test
