# Supplementary material for: Tracing feline herpesvirus-1 exposure in domestic cats from Namibia: associations with neutralising antibodies, vaccination, clinical signs, and retroviral status
Source: Vet Res Commun. 2026 Aug 5;50(5):505. doi: 10.1007/s11259-026-11446-3 (PMC13442500; doi:10.1007/s11259-026-11446-3)
Supplement: Supplementary file 1 [file 11259_2026_11446_MOESM1_ESM.docx]

**Title:** Tracing feline herpesvirus-1 exposure in domestic cats from Namibia: Associations with neutralising antibodies, vaccination, clinical signs, and retroviral status

**Journal:** Veterinary Research Communications

**Authors:** Lourens de Villiers, Simone Pulsoni, Barbara Bonfini, Lauren M. Coetzee, Ellini Hamunyela, Mari de Villiers ^e^, Eugenia Ciarrocchi ^c^, Alessandra Leone, Liana Teodori, Umberto Molini

**Corresponding author**: Department of Companion Animal Clinical Studies, Faculty of Veterinary Science, University of Pretoria, Private Bag X04, Onderstepoort, South Africa, [ldevilliers@unam.na](mailto:ldevilliers@unam.na)

**Supplementary dataset S1:** Patient and diagnostic metadata

| **Sample number** | **Region** | **Town** | **FHV -1 VNT result** | **Sex** | **Age** | **Vaccination status** | **Anorexia** | **Weight loss** | **Lethargy** | **Emaciation** | **Respiratory distress** | **Oculonasal discharge** | **Hyperthermia or pyrexia (>39.2°C)** | **Lymphadenopathy** | **Submandibular enlarged** | **FIV serology** | **FeLV serology** |
| --- | --- | --- | --- | --- | --- | --- | --- | --- | --- | --- | --- | --- | --- | --- | --- | --- | --- |
| F020 | Khomas | Windhoek | 1:16 | Female | <1 year | Vaccinated | No | No | No | No | No | No | No | No | No | Negative | Negative |
| F021 | Khomas | Windhoek | 1:32 | Female | >1 year | Unvaccinated | No | No | No | No | No | Yes | No | No | No | Negative | Negative |
| F025 | Khomas | Groot Aub | 1:4 | Male | >1 year | Unvaccinated | No | No | No | No | No | No | No | No | No | Negative | Negative |
| F029 | Khomas | Brakwater | 1:4 | Male | >1 year | Unvaccinated | No | No | No | No | No | No | No | No | No | Positive | Negative |
| F037 | Khomas | Brakwater | 1:64 | Male | >1 year | Unvaccinated | No | No | No | No | No | No | No | No | No | Negative | Positive |
| F047 | Khomas | Brakwater | 1:256 | Female | >1 year | Unvaccinated | No | No | No | No | No | Yes | No | No | No | Negative | Negative |
| F048 | Khomas | Brakwater | 1:16 | Male | >1 year | Unvaccinated | No | No | No | No | No | Yes | No | Yes | Yes | Negative | Negative |
| F051 | Khomas | Brakwater | 1:32 | Female | >1 year | Unvaccinated | No | No | No | No | No | Yes | No | No | No | Negative | Positive |
| F248 | Hardap | Rehoboth | 1:64 | Male | >1 year | Unvaccinated | No | No | No | No | No | Yes | No | No | No | Negative | Negative |
| F068 | Erongo | Walvis Bay | 1:16 | Female | >1 year | Unvaccinated | No | No | No | No | No | No | No | No | No | Negative | Negative |
| F069 | Erongo | Walvis Bay | 1:64 | Female | >1 year | Unvaccinated | No | No | No | No | No | Yes | No | Yes | Yes | Negative | Negative |
| F070 | Erongo | Walvis Bay | 1:128 | Male | >1 year | Unvaccinated | No | No | No | No | No | Yes | No | Yes | No | Negative | Positive |
| F074 | Erongo | Walvis Bay | 1:16 | Male | >1 year | Unvaccinated | No | No | No | No | No | Yes | No | No | No | Negative | Negative |
| F075 | Erongo | Walvis Bay | 1:8 | Male | >1 year | Unvaccinated | No | No | No | No | No | Yes | No | No | No | Negative | Negative |
| F076 | Erongo | Walvis Bay | 1:16 | Female | <1 year | Unvaccinated | No | No | No | No | No | Yes | No | No | No | Negative | Negative |
| F090 | Erongo | Walvis Bay | 1:4 | Male | <1 year | Unvaccinated | No | No | No | No | No | No | No | No | No | Negative | Positive |
| F091 | Erongo | Walvis Bay | 1:8 | Female | <1 year | Unvaccinated | No | No | No | No | No | No | No | No | No | Negative | Negative |
| F092 | Erongo | Walvis Bay | 1:4 | Female | <1 year | Unvaccinated | No | No | No | No | No | No | No | No | No | Negative | Negative |
